# Prognostic Significance of Comprehensive Gene Mutations and Clinical Characteristics in Adult T-Cell Acute Lymphoblastic Leukemia Based on Next-Generation Sequencing

**DOI:** 10.3389/fonc.2022.811151

**Published:** 2022-02-24

**Authors:** Hua Yin, Mei Hong, Jun Deng, Lan Yao, Chenjing Qian, Yao Teng, Tingting Li, Qiuling Wu

**Affiliations:** ^1^ Institute of Hematology, Union Hospital, Tongji Medical College, Huazhong University of Science and Technology, Wuhan, China; ^2^ Collaborative Innovation Center of Hematology, Soochow University, Suzhou, China

**Keywords:** T-cell acute lymphoblastic leukemia/lymphoma, next-generation sequencing, mutations, clinical characteristics, risk stratification

## Abstract

**Background:**

Adult T-cell acute lymphoblastic leukemia (T-ALL) is a heterogeneous malignant tumor with poor prognosis. However, accurate prognostic stratification factors are still unclear.

**Methods:**

Data from 90 adult T-cell acute lymphoblastic leukemia/lymphoma (T-ALL/LBL) patients were collected. The association of gene mutations detected by next-generation sequencing and clinical characteristics with the outcomes of T-ALL/LBL patients were retrospectively analyzed to build three novel risk stratification models through Cox proportional hazards model.

**Results:**

Forty-seven mutated genes were identified. Here, 73.3% of patients had at least one mutation, and 36.7% had ≥3 mutations. The genes with higher mutation frequency were *NOTCH1*, *FBXW7*, and *DNMT3A*. The most frequently altered signaling pathways were NOTCH pathway, transcriptional regulation pathway, and DNA methylation pathway. Age (45 years old), platelet (PLT) (50 G/L), actate dehydrogenase (LDH) (600 U/L), response in D19-BMR detection, TP53 and cell cycle signaling pathway alterations, and hematopoietic stem cell transplantation (HSCT) were integrated into a risk stratification model of event-free survival (EFS). Age (45 years old), white blood cell (WBC) count (30 G/L), response in D19-BMR detection, TP53 and cell cycle signaling pathway alterations, and HSCT were integrated into a risk stratification model of overall survival (OS). According to our risk stratification models, the 1-year EFS and OS rates in the low-risk group were significantly higher than those in the high-risk group.

**Conclusions:**

Our risk stratification models exhibited good prognostic roles in adult T-ALL/LBL patients and might guide individualized treatment and ultimately improve their outcomes.

## Introduction

T-cell acute lymphoblastic leukemia (T-ALL) in adults is an aggressive and heterogeneous hematopoietic malignancy caused by the clonal proliferation and abnormal differentiation of T lymphoid progenitor cells. Nowadays, due to the standard frontline intensive chemotherapy, 85% of T-ALL patients have achieved complete remission (CR) (1, 2). However, there is still up to 40% of adults who relapse after intensive chemotherapy, with 5-year overall survival (OS) less than 7% (3). Therefore, finding new therapeutic targets and using precisely targeted drugs are of great significance to improve the therapeutic efficacy of T-ALL.

Currently, the intensity of T-ALL treatment is based on the risk stratification using a combination of age, white blood cell (WBC) count, and extramedullary infiltration, cytogenetic, and early response to induction chemotherapy. However, it is still difficult to accurately predict the prognosis of adult T-ALL patients according to present risk stratification models. With the rapid development of next-generation sequencing (NGS) in recent years, the genomic analyses of T-ALL have been extensively explored and various genetic markers associated with T-ALL pathogenesis were identified (4–7). It has been indicated that genomic analyses could systematically identify genetic risk loci for T-ALL susceptibility (8) and support prenatal origin (9, 10). A latest study demonstrated that the mutated gene profile of adult T-ALL patients differed from that of pediatric patients and indicated an association with age in T-ALL patients (11). Furthermore, genomic analysis is conducive to comprehend the genetic basis of clonal evolution and relapse in T-ALL (12–14). A recent study also revealed that the genomic analyses can early predict the relapse of adult T-ALL driven by mutated genes and may guide clinical decisions (15). In addition, gene mutations and signaling pathway alterations based on genomic analyses are important predictors of clinical outcome in adult ALL (16). Up-to-date risk stratification of T-ALL patients based on the genome analyses showed that gene mutations had impacts on prognosis and were conducive to subdivide cases into different risk groups (17). Therefore, integration of gene mutations into current risk stratification criteria may be beneficial to improve prognosis identification and therapeutic efficacy. However, relative data are mostly lacking in adult T-ALL.

In this study, we simultaneously collected gene mutation profiles by NGS and clinical characteristics in 90 adult T-cell acute lymphoblastic leukemia/lymphoma (T-ALL/LBL) patients. Statistical analysis identified that some gene mutations were significantly correlated with clinical prognostic indicators including CR, minimal residual disease (MRD), event-free survival (EFS), relapse-free survival (RFS), and OS. Based on these prognosis-related gene mutations and clinical characteristics, we established three T-ALL risk stratification models to predict long-term prognosis and guide individualized regimens.

## Patients and Methods

### Patients and Treatment Protocol

A retrospective analysis had been conducted on 90 T-ALL/LBL patients hospitalized in Wuhan Union Hospital from June 2016 to June 2021. All patients, who were diagnosed as T-ALL/LBL according to the 2016 World Health Organization (WHO) diagnostic criteria, underwent bone marrow (BM) examinations such as cell morphology, immunophenotype, fluorescence *in situ* hybridization (FISH), fusion gene, cytogenetics, and molecular genetics (namely, NGS).

According to the Chinese guidelines (2021 version), all patients in our study received induction and intensive chemotherapy [daunorubicin, vincristine, cyclophosphamide, l-asparaginase, and prednisone (DVCLP), daunorubicin, vincristine, l-asparaginase, and prednisone (DVLP), hyper-fractionated cyclophosphamide, vincristine, doxorubicin, and dexamethasone/methotrexate, cytarabine (Hyper-CVAD/MA)]. Some T-ALL/LBL patients with suitable transplantation donors accepted hematopoietic stem cell transplantation (HSCT) after remission (if age ≤55 years old). This study has been approved by the Ethics Committee of Tongji Medical College of Huazhong University of Science and Technology and followed the principles of the Declaration of Helsinki.

### Flow Cytometry

In accordance with WHO’s guidelines, all 90 cases were diagnosed as T-ALL/LBL by particular immunophenotypic markers (usually TdT positive, usually expressing cCD3 and CD7, variably expressing CD1a, CD2, CD3, CD4, CD5, CD7, and CD8). T-ALL/LBL was further classified into pro-T-ALL, pre-T-ALL, cortical T-ALL, and medullary T-ALL according to the European Group for the Immunological Characterization of Leukemias (EGIL) classification standard (2, 18).

### Cytogenetic Analysis

Clonal karyotypes in mitotic phases were detected by G-banding chromosome analysis under microscope and were described according to the International System for Human Cytogenetic Nomenclature (ISCN, 2013).

### Next-Generation Sequencing

The mononuclear cells isolated from the newly diagnosed patients’ BM were later used for whole genome DNA (gDNA) extraction, and then NGS technology was applied to determine the type, location, and frequency of each gene mutation using a predesigned hematopoietic tumor-related hotspot gene panel (Further details of gene panels are available in the **Supplementary Appendix**). Detailed methodology was described below. The gDNA concentration was required to be ≥10 ng/μl, OD260/OD280 = 1.7–1.9, and the total mass ≥1,000 ng. The Illumina standard library (Illumina, Inc.) was then constructed and Agilent 2100 (Agilent, Inc.) was used to assess the spectrum of DNA fragments in the library, and the main peak size of the library was about 350 bp. The Roche NimbleGen liquid phase hybridization capture chip was used to target capture 214 genes with 445k in size (Roche, Inc.). QPCR quantification was carried out to measure the library concentration; the concentration of each library should be ≥10 nmol/L. PE75 sequencing was performed on Illumina Nextseq 550AR (Illumina, Inc.) after completion of the library control. Sequencing data were analyzed using the following methods: the in-house developed quality control tools were firstly used to initiate the preprocessing and quality control analysis of the raw sequencing data, followed by using the Burrows-Wheeler Alignment (BWA) algorithm to compare the processed sequencing data with the reference human genome (version: GRCh37/hg19). Picard was chosen for PCR duplication labeling, and GATK’s BaseRecalibrator was used for quality value correction of sequence alignment results. Based on the cosmic database, we used a self-built Panel of Normals (PON) with a large sample to exclude germline mutations and common single nucleotide polymorphisms (SNPs) and filter output of the variants manually. Based on the paired samples, the MuTect2 software was used for single-nucleotide variation (SNV) and Insertion/Deletion (INDEL) mutation detection, and the self-built method was used for internal tandem duplication (ITD) and protein transduction domain (PTD) mutation detection. Detection limit of NGS was set to 0.5%. Variants were annotated using Annovar software for all tests, and to ensure data quality, the average effective depth of each sample captured in the target area was required to be ≥1,000x, and it was required that all reads that support mutant types have a quality and base quality higher than 30.

### Statistical Methods

The follow-up was carried out until June 2021. OS was calculated from the date of diagnosis of T-ALL/LBL to the date of death for patients who died or the last follow-up date for those who were alive at the time of the analysis. EFS was calculated from the beginning of treatment until the date of induction failure, first relapse, or death. Response in BM was evaluated on the 19th day (D19-BMR) during induction treatment and was categorized as M1 (lymphoblasts <5%), M2 (5%–25%), and M3 (≥25%). Univariate and multivariate analyses were performed to identify potential prognostic factors. The chi-square (*X^2^*) test and Fisher’s exact test were applied to identify pairwise relationships between genetic alterations. The variables with *P* < 0.1 in univariate analysis were incorporated into the Cox proportional hazards model for multivariate analysis. CR, MRD, EFS, RFS, and OS were calculated by the Kaplan–Meier method, and then differences between groups were compared by the log-rank test.

The candidate risk factors were included into the Cox proportional hazards model and filtered by least absolute shrinkage and selection operator (LASSO) regularization. The models were checked by variance inflation factor (VIF) and C-index. All analyses were performed by R statistical software 4.0.1. A two-sided *P* < 0.05 indicated that the difference was statistically significant.

## Results

### Gene Mutational Analysis Based on Next-Generation Sequencing

#### Gene Mutation Profiles

**Figure 1:**
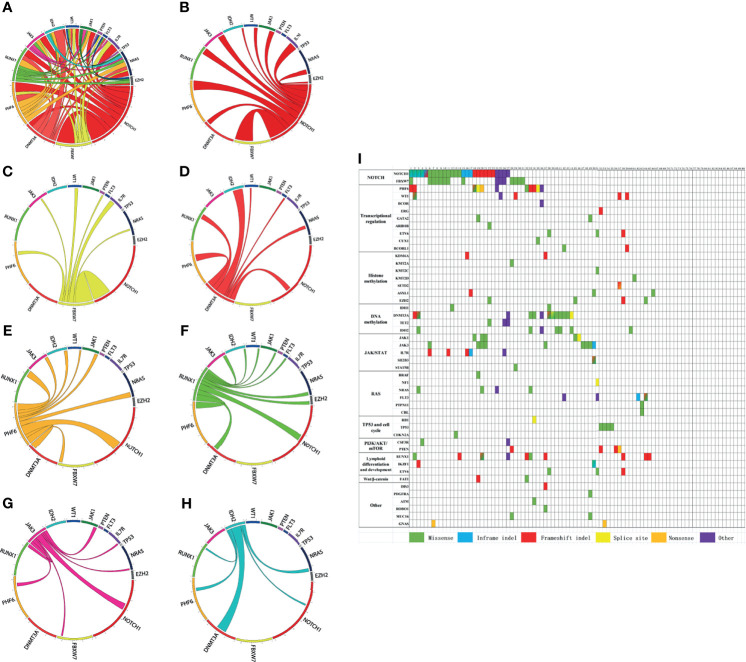
**(A–H)** Circos plots visually depict the pairwise correlation of gene mutations in our dataset. **(I)** Mutated genes are grouped by signaling pathways. The figure shows the mutational landscapes of 90 adult T-cell acute lymphoblastic leukemia/lymphoma (T-ALL/LBL) patients. Each column represents a patient, and each row represents a gene. Each color indicates a type of mutation. Blended color square denotes more than two mutation types, which are represented by the corresponding colors.

Among the 90 newly diagnosed T-ALL/LBL patients, 66 cases (73.3%) had at least 1 mutation and 33 cases (36.7%) had more than 3 mutations. There were even 2 cases with 6 mutations. The gene with the highest mutational frequency was *NOTCH1* 30.0% (27/90), followed by *FBXW7* 16.7% (15/90), *DNMT3A* 14.4% (13/90), *PHF6* 12.2% (11/90), *RUNX1* 11.1% (10/90), *JAK3* 10.0% (9/90), and *IDH2* 7.8% (7/90) (**Table S1** and **Supplementary Figure S1**). Pairwise correlations of these gene mutations in our dataset were visually depicted by Circos plots (**Figures 1A**–**H**).

Mutated genes are grouped by signaling pathways. The mutational landscapes of 90 adult T-ALL/LBL patients were described in **Figure 1I**. Signaling pathway analyses were further performed, and the most frequently altered pathway was the NOTCH pathway (34.4%, 31/90), followed by the transcriptional regulation pathway (24.4%, 22/90), DNA methylation pathway (18.9%, 17/90), Janus kinase/signal transducer and activator of transcription (JAK/STAT) pathway (18.9%, 17/90), lymphoid differentiation and development pathway (15.6%, 14/90), histone methylation pathway (14.4%, 13/90), RAS signal pathway (11.1%, 10/90), TP53 and cell cycle pathway (6.7%, 6/90), phosphatidylinositol 3-kinase/protein kinase-B/mammalian target of rapamycin (PI3K/AKT/mTOR) pathway (6.7%, 6/90), and Wnt/β-catenin pathway (2.2%, 2/90) (**Table S1**, **Supplementary Figure S1**). The frequency of other mutated genes and altered signaling pathways were shown in **Supplementary Table S1** and **Figure S1**.

#### The Pairwise Relationship Between Genetic Alterations

The pairwise analysis of all mutated genes and signal pathways were shown in **Tables S2, S3**. By integrated mutational analysis, we found significant co-occurrence of *NOTCH1* mutations and *FBXW7* mutations, *NOTCH1* mutations and *IL7R* mutations, *FBXW7* mutations and *IL7R* mutations, *PHF6* mutations and *NRAS* mutations, and *DNMT3A* mutations and *IDH2* mutations (*P* < 0.05 for all comparisons) (**Table S4**). Results also disclosed some frequently co-occurring signal pathways, including histone methylation signaling pathway and lymphoid differentiation and development signaling pathway, RAS signaling pathway and lymphoid differentiation and development signaling pathway, RAS signaling pathway and transcriptional regulation signaling pathway, lymphoid differentiation and development signaling pathway and transcriptional regulation signaling pathway, and JAK/STAT signaling pathway and NOTCH signaling pathway (*P* < 0.05 for all comparisons) (**Table S4**). No mutated genes or altered signal pathways were found mutually exclusive in our study.

#### Prognostic Value of Gene Mutations

We further analyzed the prognostic value of gene mutations (**Table S5**) and found that *FBXW7* mutations and *PTEN* mutations were related to increased CR rate (*P* < 0.001 and *P* < 0.05, respectively), while *DNMT3A* mutations were related to decreased CR rate (*P* < 0.05). However, *NOTCH*, *PHF6*, *JAK3*, and *IL7R* mutations had no significant effect on CR. Patients with *FBXW7* mutations had a significantly increased MRD negative rate (*P* = 0.006). However, no gene mutations had remarkable effects on EFS in our study. Patients with *WT1* mutations had significantly decreased RFS (*P* < 0.001). The OS of patients with *TP53* or *FLT3* mutations was significantly shortened (both *P* < 0.05), while *NOTCH1*, *FBXW7*, *IL7R*, *IDH2*, and *DNMT3A* mutations had no remarkable effects on OS.

Univariate analysis of signaling pathways (**Table S6**) showed that DNA methylation pathway alterations, TP53 and cell cycle pathway alterations, and lymphoid differentiation and development pathway alterations were related to decreased CR rate (all *P* < 0.05). DNA methylation signaling pathway alterations and lymphoid differentiation and development signaling pathway alterations were related to increased MRD positive rate (*P* < 0.05, respectively). Patients with TP53 and cell cycle signaling pathway alterations had significantly decreased EFS (*P* < 0.001), while patients with JAK/STAT pathway alterations had significantly increased EFS (*P* < 0.05). However, no signaling pathways had effects on RFS in our study. Results also indicated that the OS of patients with TP53 and cell cycle signaling pathway alterations was significantly shortened (*P* = 0.001), while the OS of patients with JAK/STAT signaling pathway alterations was significantly extended (*P* < 0.05).

### Clinical Characteristics Analysis

#### Clinical Characteristics of Patients

Besides gene mutational analysis, we also summarized the primary clinical characteristics of these 90 newly diagnosed T-ALL/LBL patients (**Table S7**). The median age was 27 years (range from 14 to 70 years old). The median follow-up time was 6 months. Here, 85.6% of patients (77/90) were diagnosed as T-ALL, while the other 13 patients were T-LBL. Additionally, according to the immunophenotype of patients, 32 of them were categorized as pro-T (35.6%), 36 as pre-T (40%), and 22 as cortical T subtype (24.4%). Furthermore, up to 46.7% cases in our study (42/90) met criteria for early T-cell precursor (ETP)-ALL according to the 2016 WHO (19). Moreover, 33 common leukemia fusion genes in our study were detected by RT-PCR (Further details of the 33 fusion genes are available in the Supplementary Appendix.).

#### Univariate Analysis of Clinical Characteristics

The clinical characteristics associated with prognostic markers including CR, MRD, EFS, RFS, and OS were screened out by univariate analysis. As shown in **Table S7**, age, immunophenotype, WT1 expression, day 8 prednisone response, and day 19 lymphoblast percentage are predictors of reaching CR rate and MRD negative rate. These and other clinical characteristics were predictors of EFS, RFS, and OS as summarized in **Table S7**. The number of cases who had a certain gene fusion in our study was slightly less (14/90), and univariate analysis showed that the fusion genes were not associated with the prognosis of adult T-ALL/LBL patients, so that fusion genes were not included in risk stratification.

### Multivariate Analysis of Gene Mutations and Clinical Characteristics

The statistically significant risk factors in gene mutations and clinical characteristics from univariate analysis above were chosen for further multivariate analysis. It revealed that Hb >100 g/L and M1 in D19-BMR detection were independent favorable prognostic factors for CR, while DNA methylation signaling pathway alterations and ETP were independent negative prognostic factors for CR. Cortical T and M1 in D19-BMR detection were independent favorable prognostic factors for MRD, while DNA methylation signaling pathway alterations were independent negative prognostic factors for MRD. Age ≤45 years old, PLT >50 G/L, LDH ≤600 U/L, HSCT, and M1+M2 in D19-BMR detection were independent favorable prognostic factors for EFS, while TP53 and cell cycle signaling pathway alterations were independent negative prognostic factors for EFS. Age ≤45 years old, WBC count ≤30 G/L, HSCT, and M1+M2 in D19-BMR detection were independent favorable prognostic factors for OS, while TP53 and cell cycle signaling pathway alterations were independent negative prognostic factors for OS. However, risk factors for RFS by univariate analysis were too few to carry out further multivariate analysis.

### Risk Stratification Models of Overall Survival in 90 Adult T-ALL/LBL Patients

**Figure 2:**
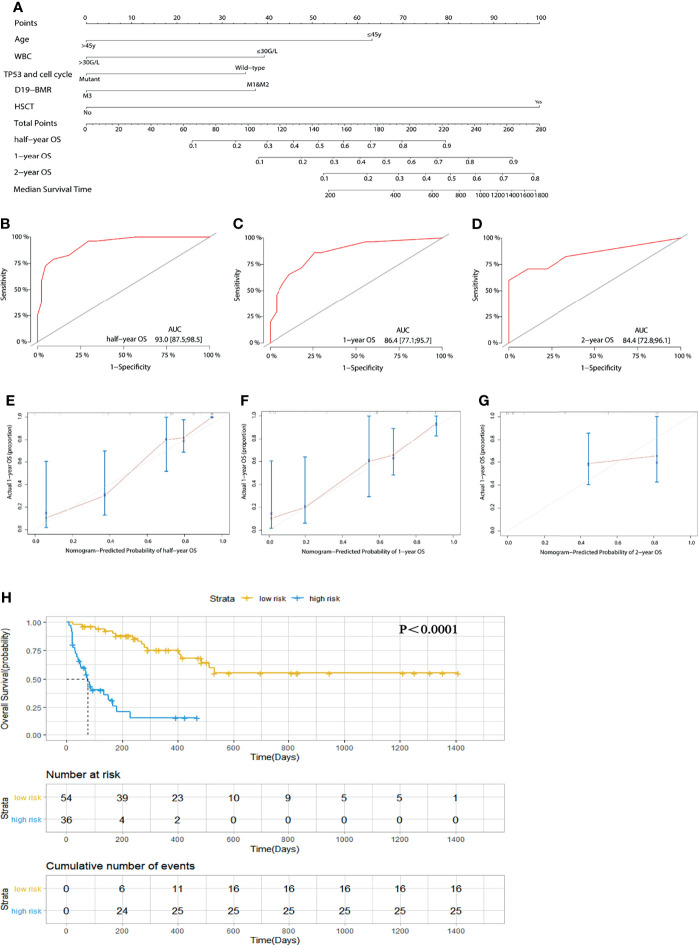
**(A)** A nomogram predicts the half-year, 1-year, and 2-year overall survival (OS) of 90 adult T-cell acute lymphoblastic leukemia/lymphoma (T-ALL/LBL) patients. **(B–D)** The AUC of nomogram for the half-year, 1-year, and 2-year OS. **(E–G)** Calibration curves for predicting half-year, 1-year, and 2-year OS. **(H)** Kaplan–Meier survival curves of OS. The diagonal gray lines could help to judge the agreement between predictions and actual observations in the AUC and calibration curves. The dotted lines drawn on the Kaplan–Meier curves were used to reveal the median survival time of patients when 50% of patients had the event. The data in the tables showed the number at risk and cumulative number of events at specific time points.

Univariate and multivariate analyses showed that age (45 years old), WBC count (30 G/L), response in D19-BMR detection, TP53 and cell cycle signaling pathway alterations, and HSCT were independent predictors for OS (**Table 1**). Then, the above five independent predictors of OS were integrated into an OS rate estimation nomogram (**Figure 2A**). The C-index of the nomogram was 0.844 (**Figures 2B**–**D**). The calibration plots showed good agreement between predictions and actual observations in our study (**Figures 2E**–**G**). In order to well evaluate the prognosis of patients, the receiver operating characteristic (ROC) analysis was conducted and the area under receiver operating characteristic curves (AUC) was calculated. The Youden Index was used to determine the optimal cutoff point that has the highest combination of sensitivity and specificity to discriminate between low-risk and high-risk patients. With the threshold score of 140 for OS nomogram, 54 patients with total points ≥140 (AUC ≥86.4) were defined as low-risk group and 36 patients <140 (AUC <86.4) as high-risk group. The 1-year OS rate of T-ALL/LBL patients in the low-risk group was significantly higher than that in the high-risk group [all patients: 70.4% vs. 30.6%, *P* < 0.0001; hazard ratio (HR): 7.956, 95% CI: 3.915–16.17] (**Figure 2H**).

**Table 1:** Univariate and multivariate analysis for OS in 90 adult T-ALL patients.

Variable	Univariate		Multivariate				
	HR (95% CI)	*P*	HR (95% CI)	*P*	c-index	vif	nomo score
Age at diagnosis (45y)	4.868 (2.438-9.721)	9.55742E-07	3.1854 (1.41962-7.1476)	0.00496	0.844	1.266289	0/63
WBC (30G/L)	1.88 (1.016-3.478)	0.04123618	2.9731 (1.50880-5.8585)	0.00164		1.168878	0/40
TP53 and cell cycle	4.28 (1.639-11.18)	0.001376429	3.0074 (1.12213-8.0603)	0.02859		1.017995	0/35
Response in D19-BMR detection (M1+M2/M3)	3.407 (1.823-6.367)	4.74972E-05	2.1497 (1.10235-4.1923)	0.02471		1.093628	0/37
HSCT	0.1537 (0.07346-0.3218)	4.99E-08	0.1764 (0.07721-0.4029)	3.84E-05		1.134547	0/100

Of these 90 adult T-ALL/LBL patients, 39 patients received HSCT after chemotherapy. The median follow-up time after HSCT was 153 days (range from 23 to 1,200 days). Among them, 13 patients relapsed after HSCT. The cumulative incidence rate (CIR) was 33.3% (13/39), and the non-relapse mortality (NRM) was 3.8% (1/26) (**Supplementary Figure S2**). The median follow-up time of leukemia-free survival was 233 days (range from 23 to 1,200 days).

**Figure 3:**
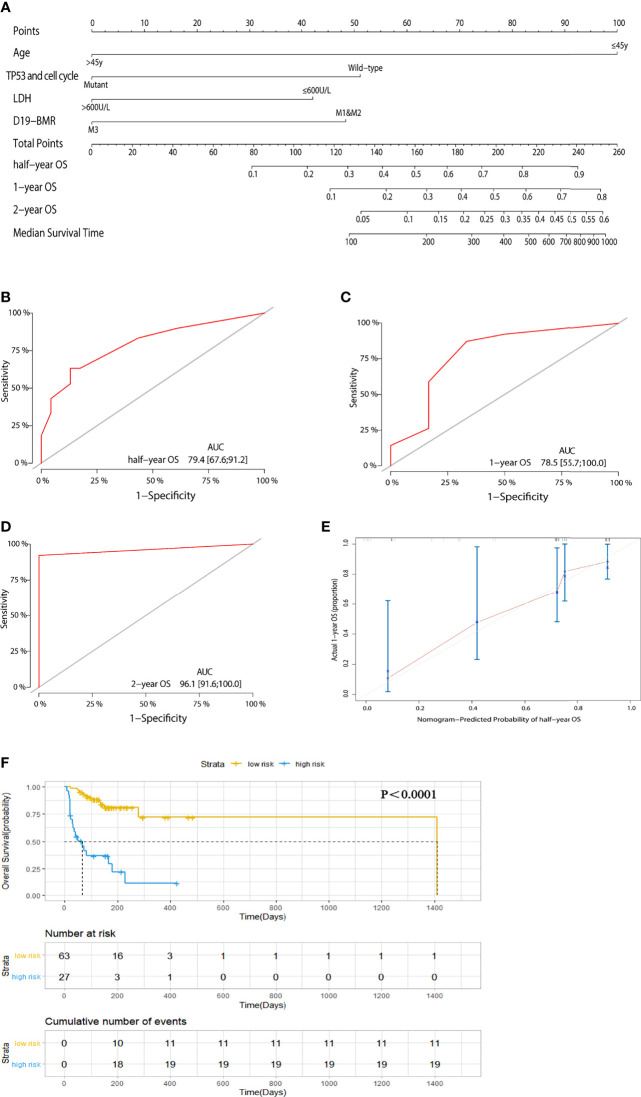
**(A)** A nomogram predicts the half-year, 1-year, and 2-year overall survival (OS) of 90 adult T-cell acute lymphoblastic leukemia/lymphoma (T-ALL/LBL) patients while removing the impact of hematopoietic stem cell transplantation (HSCT). **(B–D)** The AUC of nomogram for the half-year, 1-year, and 2-year OS. **(E)** Calibration curves for predicting 1-year OS. **(F)** Kaplan–Meier survival curves of OS.

In order to remove the impact of HSCT on the prognosis for patients, we adopted “censored data” to process the transplantation data and then built a new risk stratification model for OS in 90 adult patients. Univariate and multivariate analyses showed that age (45 years old), LDH (600 U/L), response in D19-BMR detection, and TP53 and cell cycle signaling pathway alterations were independent predictors for OS (**Table 2**). The new risk stratification model of OS was also built into a nomogram (**Figure 3A**). The C-index of the nomogram was 0.792 (**Figures 3B**–**D**). The calibration plots also showed good agreement between predictions and actual observations in our study (**Figure 3E**). With the threshold score of 170 for OS nomogram, 27 patients with total points ≥170 (AUC ≥78.5) was defined as low-risk groups and 63 patients <170 (AUC <78.5) as high-risk groups. The 1-year OS rate of T-ALL/LBL patients in the low-risk group was significantly better than that in the high-risk group (69.6% vs. 21.7%, *P* < 0.00019; HR: 3.8, 95% CI: 1.803–8.01) (**Figure 3F**).

**Table 2:** Univariate and multivariate analysis for OS in 90 patients removing the impact of HSCT.

Variable	Univariate		Multivariate				
	HR (95% CI)	*P*	HR (95% CI)	*P*	c-index	vif	nomo score
Age at diagnosis (45y)	7.087 (3.332-15.07)	3.66E-07	8.018 (3.272-19.649)	5.32E-06	0.792	1.237466	0/100
TP53 and cell cycle	4.464 (1.69-11.79)	2.53E-03	4.294 (1.558-11.834)	0.00484		1.015417	0/51
LDH (600U/L)	1.803 (0.8758-3.711)	0.11000	3.630 (1.599-8.237)	0.00205		1.248115	0/42
Response in D19-BMR detection (M1+M2/M3)	3.78 (1.814-7.877)	3.85E-04	3.185 (1.440-7.045)	0.00422		1.090931	0/48

### Risk Stratification Model of Event-Free Survival in 90 Adult T-ALL/LBL Patients

Univariate and multivariate analyses showed that age (45 years old), PLT (50 G/L), LDH (600 U/L), response in D19-BMR detection, TP53 and cell cycle signaling pathway alterations, and HSCT were independent predictors for EFS (**Table S8**). Then, the above six independent predictors of EFS were integrated into the nomogram of estimating EFS rate with the C-index 0.844 (**Supplementary Figures S3A**–**D**). The calibration plots also showed good consistency between predictions and actual data (**Supplementary Figures S3E**–**G**). With the threshold score of 150, 58 patients with total points ≥150 (AUC ≥85.4) belonged to the low-risk group and 32 patients <150 (AUC <85.4) belonged to the high-risk group. The 1-year EFS rate of T-ALL/LBL patients in the low-risk group was significantly higher than that in the high-risk group (all patients: 67.2% vs. 25.0%, *P* < 0.0001; HR: 7.002, 95% CI: 3.642–13.46) (**Supplementary Figure S3H**).

## Discussion

To date, there is still a lack of universally accepted criteria combining gene mutations with clinical characteristics for T-ALL risk stratification. Therefore, in this study, we established three novel risk stratification models by the combination of gene mutations and clinical characteristics with EFS and OS to predict therapeutic efficacy and prognosis in adult T-ALL/LBL patients, which displayed favorable predictive efficacy. One latest study involving genomic analyses of ALL by copy number alteration (CNA) profiling indicated that 8 genes (*IKZF1*, *CDKN2A/2B*, *PAR1*, *BTG1*, *EBF1*, *PAX5*, *ETV6*, and *RB1*) had potential to serve as risk stratification markers (20), which partly overlapped with our results about gene mutations, indicating the applicability of our study.

*TP53* is a typical tumor suppressor gene. *TP53* mutation is involved in the pathogenesis of various tumors, including T-ALL. The frequency of *TP53* mutations in newly diagnosed T-ALL in our study was slightly higher than previously reported (4.4% vs. 2%–3%) (21). In Pediatric Oncology Group protocol POG8862, *TP53* mutations usually occurred in relapsed T-ALL children, who had a worse survival than children without *TP53* mutations (22). In addition, *TP53* mutations were found associated with worse 5-year EFS and OS (23), which was consistent with our results. In our study, TP53 pathway alteration is an independent unfavorable risk factor for EFS and OS. Besides, the OS in the patients with *TP53* mutations was significantly shortened, whose median survival time was 53 days.

*DNMT3A* mutations frequently occur in myeloid tumors but are less common in lymphoid malignancies that are mainly found in T-cell lineage diseases (24, 25). Besides, the mutation frequency of *DNMT3A* increased with age and was extremely rare in children and adolescents with T-ALL (25). Mutation frequency of *DNMT3A* in our study was 14.4%, which was higher than previously reported, 9.1% (25), but lower than previously reported, 17.8% (26). Previous studies demonstrated that *DNMT3A* mutations were significantly associated with shorter EFS and OS, which were independent prognostic factors for EFS but not OS (25). Another study from MRC UKALL XII/ECOG E2993 reported that *DNMT3A* was an independent prognostic marker in adult T-ALL that might be useful for risk stratification of high-risk early immature adult T-ALL (27). In our study, the median times of reaching both CR and MRD in patients with *DNMT3A* mutations are much longer than those of patients without *DNMT3A* mutations. Furthermore, DNMT3A pathway alteration is an independent unfavorable risk factor for CR and MRD, which suggested that the patients with *DNMT3A* mutations and DNA methylation signaling pathway alterations have worse early response to chemotherapy. Decitabine, a DNA hypomethylating agent, was reported to be a promising therapeutic agent for relapsed ALL after HSCT (28). Besides, a patient with relapsed T-ALL after HSCT achieved an effective response to the combined treatment of decitabine and venetoclax (29). So, hypomethylating agent combined with chemotherapy might be recommended for T-ALL patients with *DNMT3A* mutations and DNMT3A pathway alterations to increase the CR rate.

*NOTCH1* was a class I transmembrane glycoprotein that functions as a ligand-activated transcription factor, directly transducing extracellular signals on the cell membrane and triggering the expression of specific target genes in the nucleus (30). Activation of NOTCH signaling pathway by *NOTCH1* and/or *FBXW7* mutations was a prominent oncogenic event in the hematopoietic system, also critical for the development of T cells and the regulation of many important cellular processes. In our study, the mutation frequency of *NOTCH1* (30%) was lower than the previously reported 45.8%–66% (16, 31–33). The mutation frequency of *FBXW7* was between the reported data 18% (31) and 9.4% (32). The mutation frequency of *NOTCH* signaling pathway was lower than reported data 59%–73.3% (27, 34–36). The role of *NOTCH1* mutations in T-ALL is still controversial. Our study showed that *NOTCH1* mutations have no significant impact on CR, MRD, EFS, RFS, and OS, which was completely consistent with results of some studies (31, 33, 35, 37). However, some researchers reported that T-ALL patients with *NOTCH1*/*FBXW7* mutations had better OS when compared with wild-type cases (5, 27, 37), and *NOTCH1* mutations predicted a faster early treatment response (38). Apart from the favorable role, Zhu et al. (39) reported that *NOTCH1* mutations were relevant to shorter OS in T-ALL patients. Therefore, a larger sample size is needed for the confirmation of the role of *NOTCH1* mutations.

In this study, we also identified the pairwise relationship between genetic alterations and found significant co-occurrence of *NOTCH1* mutations and *FBXW7* mutations, *NOTCH1* mutations and *IL7R* mutations, *FBXW7* mutations and *IL7R* mutations, *PHF6* mutations and *NRAS* mutations, and *DNMT3A* mutations and *IDH2* mutations. Of note, T-ALL is a genomically heterogeneous malignancy as discussed, and co-occurrence of specific mutations could contribute to leukemogenesis (13). Preclinical studies suggest that co-occurring mutations may impact treatment responsiveness, since the treatment response to docetaxel monotherapy in lung tumors was markedly impaired when *KRAS* mutants co-occurred with *TP53* mutations (40). Furthermore, *KRAS* mutations co-occurring with *TP53* mutations are associated with increased intratumoral T-cell infiltration, programmed cell death protein (*PD-1*) expression, and prolonged clinical benefit from anti-PD-1 immunotherapy in non-small cell lung cancer (NSCLC) (41). Although *IDH1* and *IDH2* both regulate DNA methylation, mutations to *IDH1* and *IDH2* are mutually exclusive (42), which was also observed in our study. Furthermore, it has been reported that *IDH1* and *IDH2* mutations are frequently co-occurring with *DNMT3A* mutations in AML. In particular, the prognosis was significantly worse for the co-occurrence of *DNMT3A* mutations with *IDH2* mutations (43). In addition, it has been reported that *DNMT3A*, *IDH1*, and *IDH2* mutations were uniquely present in the early immature adult T-ALL and conferred worse prognosis in adult T-ALL (27), which is consistent with our study. Some previous studies revealed that *NOTCH1/FBXW7* mutations co-occurred (44) and were significant favorable prognostic predictors for OS in adult T-ALL patients in the absence of *K/NRAS* mutation or *PTEN* mutations (45). Moreover, it has been demonstrated that JAK/STAT signaling pathway alterations were co-occurring with alterations of NOTCH signaling pathway (46, 47) and *PHF6* mutations but not with *K/NRAS*, and this population may not benefit from HSCT (46). It has been demonstrated experimentally that *PHF6* loss can enhance the oncogenic activity of *NOTCH1* mutations; therefore, *PHF6* and *NOTCH1* co-mutation are more tightly linked to T-ALL pathogenesis and leukemia-associated mortality (48, 49). Several studies demonstrated that *IL7R* mutations may be oncogenic drivers in ETP-ALL (50, 51) and positively correlated with *PHF6* mutations in the development of T-ALL (52). Interestingly, it has been observed that *PTPN2* deletions were co-occurring with alterations of IL7R/JAK-STAT signaling pathway and inclined to associate with improved OS in children, but not in adults in a large cohort of 430 adult T-ALL patients (53). Hence, co-occurring mutations may account for the limited activity of single targeted agent. Rational combination therapies are of great promise to provide precise and effective long-term disease control or remission.

The incidence of ETP-ALL gradually increased with age, which was 5.5%–13% in children (54, 55) and 30%–50% in adults (56–58). The incidence of adult ETP-ALL in our data was 46.7%. These differences may attribute to ethnic variations and demographic structure. The average age of ETP-ALL patients in this study was 37.5 years old, higher than 32 as previously reported (59). ETP-ALL has been found related to unfavorable prognosis because of poor response to chemotherapy and high relapse rate (54, 55, 60, 61). The 10-year OS for ETP-ALL was only 19% (54). However, a recent research found that not all patients with ETP-ALL had worse prognosis (62). It has been also reported that patients with ETP-ALL seemed to have an intermediate risk outcome and might have a similar prognosis compared with typical T-ALL patients if receiving intense treatment (63). In this study, ETP-ALL was an independent poor prognostic factor for CR and MRD but did not impact long-term outcomes such as EFS, RFS, and OS, which indicated that ETP-ALL was not the strictly independent factor for all prognostic markers.

Some current pediatric risk stratification models include MRD status of patients (64). In adult T-ALL, MRD ≥10^−4^ is associated with higher recurrence rate and decreased OS, which has been included in criteria for high-risk patients (16). In our study, T-ALL/LBL patients with detectable MRD had worse EFS and OS. But we found that MRD is not an independent risk factor for EFS and OS. Actually, adult ALL patients show greater heterogeneity than pediatric patients. Moreover, PCR- and flow cytometry-based MRD assessment has limited sensitivity. Standardization of methodologies and harmonization of terminology are still lacking for MRD diagnostics. These are probably the reason why MRD status has not been implemented in the risk stratification of adult T-ALL/LBL. Hence, improved detection methods and larger sample size are necessary for further validation.

It is increasingly important to accurately stratify patients who benefit from HSCT. A meta-analysis including 2,962 patients have shown a survival benefit for HSCT for patients <35 years old but not for those >35 years (65). In addition, 1,646 adults diagnosed with standard-risk or high-risk ALL in the Medical Research Council (MRC) UKALL XII/ECOG 2993 have shown superiority of HSCT on the prognosis (66). The consensus from the Chinese Society of Hematology has also recommended that HSCT is the standard of care for adult ALL patients at either standard risk or high risk who receive adult chemotherapy regimens (67). In our study, the HSCT was an independent favorable predictor for EFS and OS.

The independent risk factors we included in our risk stratification models are different from all previous models mainly because we emphasized gene mutations detected by NGS. The integration of gene mutations and clinical characteristics of adult T-ALL/LBL patients improved our understanding of their clinicobiological features, optimized the current prognostic-related risk stratification models, and provided a foundation for formulating treatment regimens. However, its limitations also deserve commentary. This was a non-randomized retrospective analysis with some potential biases. In addition, the number of cases in this study was slightly less, so that comprehensiveness of the results is limited. Therefore, it is necessary to recruit more patients and prolong follow-up time in the subsequent project to confirm the validity of our risk stratification models on adult T-ALL treatment decisions and prognosis.

## Data Availability Statement

The NGS data have been deposited in public, community supported repository. The name of the repository and accession number can be found below: Genome Sequence Archive in National Genomics Data Center and accession number HRA001815 that are publicly accessible at https://bigd.big.ac.cn/gsa-human/browse/HRA001815. Other related data are available on personal request through the corresponding author (QW) and will be made available after approval of HY, MH, and JD, who created and maintain the database.

## Ethics Statement

The studies involving human participants were reviewed and approved by the Ethics Committee of Tongji Medical College of Huazhong University of Science and Technology. Written informed consent to participate in this study was provided by the participants’ legal guardian/next of kin.

## Author Contributions

QW conceived and designed the study. HY, MH, JD, LY, CQ, YT, and TL collected and analyzed data. HY, MH, and JD wrote the paper. These three authors have contributed equally to this work and share first authorship. QW reviewed and edited the article. All authors read and approved the article.

## Funding

This study was supported by the National Natural Science Foundation of China (no. 81570193 and no. 81770219 for QW).

## Conflict of Interest

The authors declare that the research was conducted in the absence of any commercial or financial relationships that could be construed as a potential conflict of interest.

## Publisher’s Note

All claims expressed in this article are solely those of the authors and do not necessarily represent those of their affiliated organizations, or those of the publisher, the editors and the reviewers. Any product that may be evaluated in this article, or claim that may be made by its manufacturer, is not guaranteed or endorsed by the publisher.

## Supplementary Material

Supplementary Figure S1
**(A)** Frequency of mutated genes in our dataset. **(B)** Frequency of altered signaling pathways in our dataset.

Supplementary Figure S2
**(A)** The cumulative incidence rate (CIR) of the 39 patients received HSCT after chemotherapy. **(B)** The non-relapse mortality (NRM) of the 39 patients received HSCT after chemotherapy.

Supplementary Figure S3
**(A)** A nomogram predicts the half-year, 1-year and 2-year EFS of 90 adult T-ALL/LBL patients. **(B–D)** The AUC of nomogram for the half-year, 1-year and 2-year EFS. **(E–G)** Calibration curves for predicting half-year, 1-year and 2-year EFS. **(H)** Kaplan-Meier survival curves of EFS.
