# Supplementary figures and images for: Prognostic Significance of Comprehensive Gene Mutations and Clinical Characteristics in Adult T-Cell Acute Lymphoblastic Leukemia Based on Next-Generation Sequencing
Source: Front Oncol. 2022 Feb 24;12:811151. doi: 10.3389/fonc.2022.811151 (PMC8908046; doi:10.3389/fonc.2022.811151)

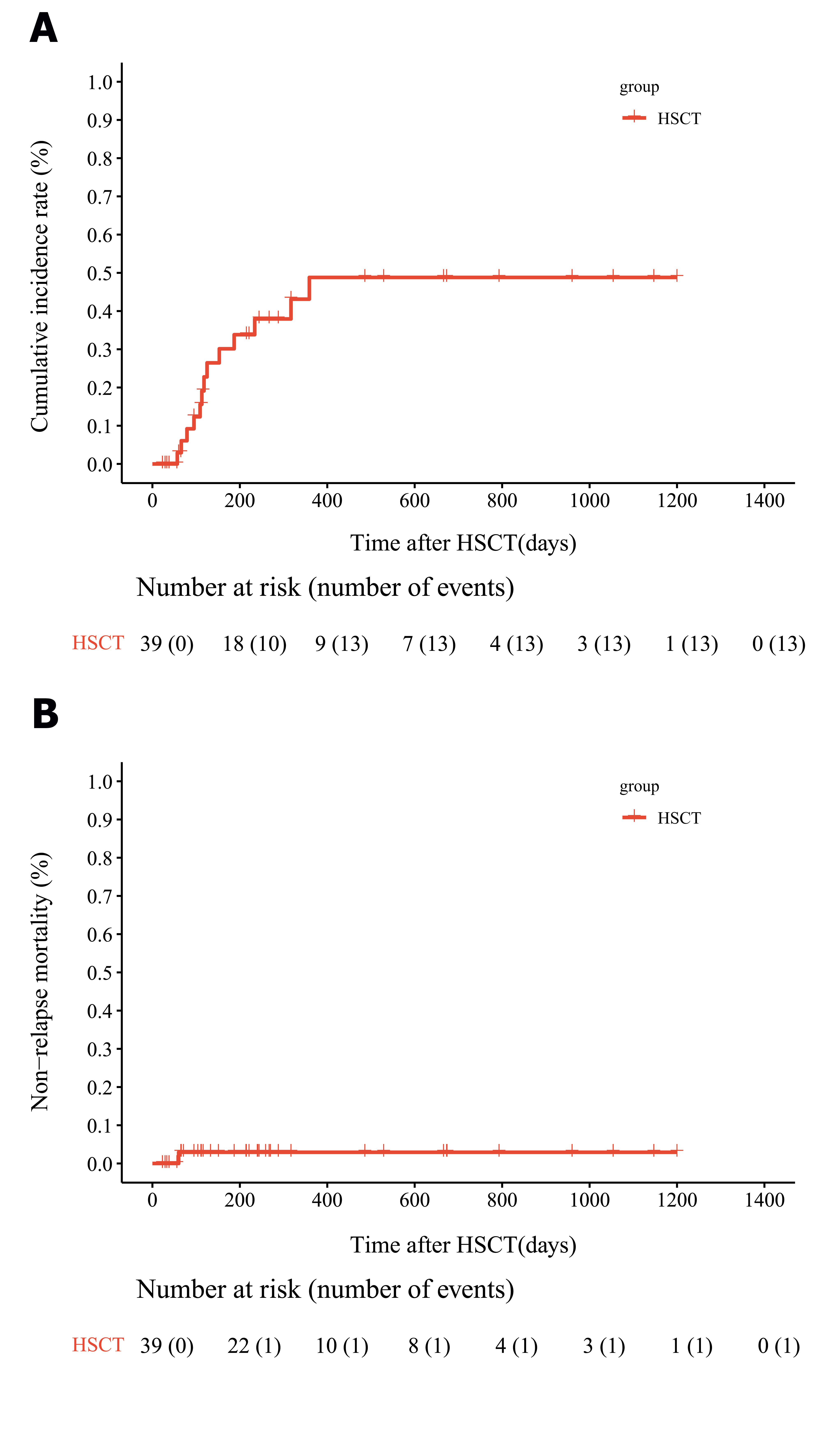

Supplement: Supplementary Figure S2 — (A) The cumulative incidence rate (CIR) of the 39 patients received HSCT after chemotherapy. (B) The non-relapse mortality (NRM) of the 39 patients received HSCT after chemotherapy. [file Image_2.jpeg]
