# Supplementary material for: Prognostic Significance of Comprehensive Gene Mutations and Clinical Characteristics in Adult T-Cell Acute Lymphoblastic Leukemia Based on Next-Generation Sequencing
Source: Front Oncol. 2022 Feb 24;12:811151. doi: 10.3389/fonc.2022.811151 (PMC8908046; doi:10.3389/fonc.2022.811151)
Supplement: Supplementary file 4 [file Table_1.docx]

**Table S1. Mutational frequency of genes and altered signaling pathways in 90 adult T-ALL patients.**

| **Gene** | **Mutant (n)** | **Overall Frequency (%)** |
| --- | --- | --- |
| NOTCH1 | 27 | 30.00% |
| FBXW7 | 15 | 16.67% |
| DNMT3A | 13 | 14.44% |
| PHF6 | 11 | 12.22% |
| RUNX1 | 10 | 11.11% |
| JAK3 | 9 | 10.00% |
| IDH2 | 7 | 7.78% |
| IL7R | 5 | 5.56% |
| WT1 | 5 | 5.56% |
| JAK1 | 5 | 5.56% |
| PTEN | 4 | 4.44% |
| FLT3 | 4 | 4.44% |
| TP53 | 4 | 4.44% |
| NRAS | 4 | 4.44% |
| EZH2 | 4 | 4.44% |
| ASXL1 | 3 | 3.33% |
| TET2 | 3 | 3.33% |
| ETV6 | 3 | 3.33% |
| KDM6A | 2 | 2.22% |
| MUC16 | 2 | 2.22% |
| CSF3R | 2 | 2.22% |
| GATA2 | 2 | 2.22% |
| IKZF1 | 2 | 2.22% |
| IDH1 | 2 | 2.22% |
| BCORL1 | 2 | 2.22% |
| FAT1 | 2 | 2.22% |
| GNAS | 2 | 2.22% |
| ATM | 1 | 1.11% |
| BCOR | 1 | 1.11% |
| CBL | 1 | 1.11% |
| CDKN2A | 1 | 1.11% |
| DIS3 | 1 | 1.11% |
| ERG | 1 | 1.11% |
| KMT2A | 1 | 1.11% |
| PDGFRA | 1 | 1.11% |
| PTPN11 | 1 | 1.11% |
| RB1 | 1 | 1.11% |
| ROBO1 | 1 | 1.11% |
| STAT5B | 1 | 1.11% |
| KMT2D | 1 | 1.11% |
| ARID1B | 1 | 1.11% |
| KMT2C | 1 | 1.11% |
| NF1 | 1 | 1.11% |
| CUX1 | 1 | 1.11% |
| BRAF | 1 | 1.11% |
| SH2B3 | 1 | 1.11% |
| SETD2 | 1 | 1.11% |

| **Signaling pathway** | **Mutant (n)** | **Overall Frequency (%)** |
| --- | --- | --- |
| NOTCH | 31 | 34.44% |
| Transcriptional regulation | 22 | 24.44% |
| DNA methylation | 17 | 18.89% |
| JAK/STAT | 17 | 18.89% |
| Lymphoid differentiation and development | 14 | 15.56% |
| Histone methylation | 13 | 14.44% |
| RAS | 10 | 11.11% |
| TP53 and cell cycle | 6 | 6.67% |
| PI3K/AKT/mTOR | 6 | 6.67% |
| Other | 6 | 6.67% |
| Wnt/β-catenin | 2 | 2.22% |
