# Supplementary material for: Prognostic Significance of Comprehensive Gene Mutations and Clinical Characteristics in Adult T-Cell Acute Lymphoblastic Leukemia Based on Next-Generation Sequencing
Source: Front Oncol. 2022 Feb 24;12:811151. doi: 10.3389/fonc.2022.811151 (PMC8908046; doi:10.3389/fonc.2022.811151)
Supplement: Supplementary file 6 [file Table_3.docx]

**Table S3. Co-occurrences of altered signaling pathways in 90 adult T-ALL patients.**

|  | DNA methylation | Histone methylation | RAS | JAK/STAT | NOTCH | TP53 and cell cycle | PI3K/AKT/mTOR | Wnt/β-catenin | Lymphoid differentiation and development | Transcriptional regulation | Other |
| --- | --- | --- | --- | --- | --- | --- | --- | --- | --- | --- | --- |
| DNA methylation |  | 1 | 3 | 1 | 6 | 1 | 1 | 0 | 5 | 7 | 1 |
| Histone methylation | 1 |  | 2 | 5 | 4 | 0 | 2 | 0 | 5 | 4 | 2 |
| RAS | 3 | 2 |  | 2 | 4 | 0 | 0 | 1 | 5 | 6 | 0 |
| JAK/STAT | 1 | 5 | 2 |  | 10 | 0 | 0 | 1 | 3 | 5 | 2 |
| NOTCH | 6 | 4 | 4 | 10 |  | 1 | 3 | 1 | 5 | 9 | 2 |
| TP53 and cell cycle | 1 | 0 | 0 | 0 | 1 |  | 1 | 0 | 0 | 2 | 2 |
| PI3K/AKT/mTOR | 1 | 2 | 0 | 0 | 3 | 1 |  | 0 | 1 | 2 | 1 |
| Wnt/β-catenin | 0 | 0 | 1 | 1 | 1 | 0 | 0 |  | 1 | 2 | 0 |
| Lymphoid differentiation and development | 5 | 5 | 5 | 3 | 5 | 0 | 1 | 1 |  | 7 | 1 |
| Transcriptional regulation | 7 | 4 | 6 | 5 | 9 | 2 | 2 | 2 | 7 |  | 2 |
| Other | 1 | 2 | 0 | 2 | 2 | 2 | 1 | 0 | 1 | 2 |  |
