# Supplementary material for: Prognostic Significance of Comprehensive Gene Mutations and Clinical Characteristics in Adult T-Cell Acute Lymphoblastic Leukemia Based on Next-Generation Sequencing
Source: Front Oncol. 2022 Feb 24;12:811151. doi: 10.3389/fonc.2022.811151 (PMC8908046; doi:10.3389/fonc.2022.811151)
Supplement: Supplementary file 7 [file Table_4.docx]

**Table S4-1. Frequently co-occurring genetic abnormalities.**

| Mutated Gene #1 | Mutated Gene #2 | M/M^1^ | WT/M^2^ | %  M/M^3^ | M/WT^4^ | WT/WT^5^ | % M/WT^6^ | *p*-value^7^ |  |
| --- | --- | --- | --- | --- | --- | --- | --- | --- | --- |
| NOTCH1 | FBXW7 | 11 | 4 | 73.3 | 16 | 59 | 21.3 | 0.0001855 | |
| NOTCH1 | IL7R | 5 | 0 | 1 | 22 | 63 | 25.9 | 0.001837 | |
| DNMT3A | IDH2 | 6 | 1 | 85.7 | 7 | 76 | 8.4 | 0.00001791 | |
| PHF6 | NRAS | 3 | 1 | 75 | 1 | 78 | 1.3 | 0.005231 | |
| FBXW7 | IL7R | 3 | 2 | 60 | 12 | 73 | 14.1 | 0.03113 | |

**Table S4-2. Frequently co-occurring signaling pathway alterations.**

| Signal Pathway #1 | Signal Pathway #2 | M/M | WT/M | %  M/M | M/WT | WT/WT | %  M/WT | *p*-value |
| --- | --- | --- | --- | --- | --- | --- | --- | --- |
| Histone methylation | Lymphoid differentiation and development | 5 | 9 | 35.7 | 8 | 68 | 10.5 | 0.02746 |
| RAS | Lymphoid differentiation and development | 5 | 9 | 35.7 | 5 | 71 | 6.6 | 0.007183 |
| RAS | Transcriptional regulation | 6 | 16 | 27.3 | 4 | 64 | 5.9 | 0.01225 |
| Lymphoid differentiation and development | Transcriptional regulation | 7 | 15 | 31.8 | 7 | 61 | 10.3 | 0.03643 |
| JAK/STAT | NOTCH | 10 | 21 | 32.3 | 7 | 52 | 11.9 | 0.02508 |

1. Number of patients mutated for both gene #1 and gene #2.
2. Number of patients wild-type for gene #1 but mutated for gene #2.
3. Percentage of patients mutant for gene #1 and gene #2 over all patients mutated for either gene.
4. Number of patients mutated for both gene #1 and wild-type for gene #2.
5. Number of patients wild-type for gene #1 and gene #2.
6. Percentage of patients mutant for either gene over all patients wild-type for either gene.
7. *P*-value by Fisher’s exact test and adjusted.
