# Supplementary material for: Prognostic Significance of Comprehensive Gene Mutations and Clinical Characteristics in Adult T-Cell Acute Lymphoblastic Leukemia Based on Next-Generation Sequencing
Source: Front Oncol. 2022 Feb 24;12:811151. doi: 10.3389/fonc.2022.811151 (PMC8908046; doi:10.3389/fonc.2022.811151)
Supplement: Supplementary file 11 [file Table_8.docx]

**Table S8. Univariate and multivariate analysis for EFS in 90 adult T-ALL patients.**

| Variable | Univariate | | Multivariate | | | | |
| --- | --- | --- | --- | --- | --- | --- | --- |
|  | HR (95% CI) | *p* | HR (95% CI) | *p* | c-index | vif | nomo score |
| Age at diagnosis (45y) | 4.629 (2.333-9.187) | 1.93436E-06 | 3.4278 (1.52144-7.7227) | 0.00295 | 0.844 | 1.332147 | 0/54 |
| PLT (50G/L) | 0.5903 (0.3239-1.076) | 0.08307071 | 0.4786 (0.24216-0.9460) | 0.03404 |  | 1.266586 | 0/28 |
| TP53 and cell cycle | 4.787 (1.833-12.510) | 0.001376429 | 5.0124 (1.75287-14.3331) | 0.00264 |  | 1.039792 | 0/48 |
| LDH(600U/L) | 1.864 (1.022-3.402) | 0.03899369 | 2.4791 (1.20598-5.0961) | 0.01353 |  | 1.387317 | 0/30 |
| Response in D19-BMR detection (M1+M2/M3) | 3.286 (1.790-6.032) | 5.21743E-05 | 2.3951 (1.18632-4.8354) | 0.01483 |  | 1.272317 | 0/40 |
| HSCT | 0.1422 (0.06836-0.2957) | 8.82E-09 | 0.1914 (0.08453-0.4332) | 0.000073 |  | 1.132811 | 0/100 |
