# Supplementary material for: Prognostic Significance of Comprehensive Gene Mutations and Clinical Characteristics in Adult T-Cell Acute Lymphoblastic Leukemia Based on Next-Generation Sequencing
Source: Front Oncol. 2022 Feb 24;12:811151. doi: 10.3389/fonc.2022.811151 (PMC8908046; doi:10.3389/fonc.2022.811151)
Supplement: Supplementary file 12 [file Table_9.docx]

214 genes covered by a pre-designed hematopoietic tumor-related

NGS hotspot gene panel

| **ABL1** | **ANKRD26** | **ASXL1** | **ASXL2** | **ATG2B** | **ATM** | **BCOR** | **BIRC3** |
| --- | --- | --- | --- | --- | --- | --- | --- |
| **BLM** | **BRAF** | **BRCA1** | **BRCA2** | **CALR** | **CBL** | **CDKN1A** | **CDKN2A** |
| **CDKN2B** | **CEBPA** | **CSF3R** | **DDX41** | **DHX15** | **DKC1** | **DNMT3A** | **ELANE** |
| **EP300** | **EPCAM** | **ETNK1** | **ETV6** | **EZH2** | **FBXW7** | **FLT3** | **FLT3-ITD** |
| **GATA1** | **GATA2** | **GATA3** | **GFI1** | **GSKIP** | **HAX1** | **IDH1** | **IDH2** |
| **IKZF1** | **IL7R** | **JAK1** | **JAK2** | **JAK3** | **KDM6A** | **KIT** | **MLL** |
| **KRAS** | **MBD4** | **MLH1** | **MPL** | **MSH2** | **MSH6** | **MYD88** | **NF1** |
| **NOTCH1** | **NPM1** | **NRAS** | **PAX5** | **PHF6** | **PIGA** | **PMS2** | **PPM1D** |
| **PRPF8** | **PTPN11** | **RAD21** | **RB1** | **RUNX1** | **SAMD9** | **SAMD9L** | **SETBP1** |
| **SF3B1** | **SH2B3** | **SMC1A** | **SMC3** | **SRP72** | **SRSF2** | **STAG2** | **STAT3** |
| **TERC** | **TERT** | **TET2** | **TP53** | **TPMT** | **TYK2** | **U2AF1** | **WT1** |
| **ZBTB7A** | **ZRSR2** | **ABCB1** | **ABCC3** | **ABL2** | **ACD** | **AKT2** | **AKT3** |
| **AMER1** | **APC** | **ARID5B** | **ATRX** | **BCL2** | **BCORL1** | **BRIP1** | **CACNA1E** |
| **CARD11** | **CBLB** | **CBLC** | **CCND1** | **CD33** | **CD79B** | **CDA** | **CEBPE** |
| **CHEK2** | **CREBBP** | **CRLF2** | **CSF1R** | **CTC1** | **CTLA4** | **CUX1** | **CYP2C19** |
| **CYP3A4** | **DIS3** | **DNAH9** | **DNAJC21** | **EBF1** | **EFL1** | **EGFR** | **EPOR** |
| **ERCC1** | **ERCC4** | **ERG** | **FAM46C** | **FANCA** | **FANCB** | **FANCC** | **FANCD2** |
| **FANCE** | **FANCF** | **FANCG** | **FANCI** | **FANCL** | **FANCM** | **FAT1** | **FGFR1** |
| **G6PC3** | **GNAS** | **GSTM1** | **GSTP1** | **HRAS** | **ID3** | **KMT2C** | **KMT2D** |
| **MAD2L2** | **MAP2K4** | **MAP3K7** | **MDM2** | **MEF2B** | **MTHFR** | **NAF1** | **NF2** |
| **NHP2** | **NOP10** | **NOTCH2** | **NQO1** | **NSD2** | **NT5C2** | **NTRK1** | **NTRK2** |
| **NTRK3** | **PALB2** | **PARN** | **PDGFRA** | **PDGFRB** | **PIGT** | **PIK3CA** | **POT1** |
| **PRPS1** | **PTEN** | **RAD51** | **RAD51C** | **RPL11** | **RPL15** | **RPL23** | **RPL26** |
| **RPL27** | **RPL31** | **RPL35A** | **RPL5** | **RPS10** | **RPS17** | **RPS19** | **RPS24** |
| **RPS26** | **RPS27** | **RPS28** | **RPS29** | **RPS7** | **RTEL1** | **SBDS** | **SETD2** |
| **SF1** | **SLX4** | **SMAD4** | **STAT5A** | **STAT5B** | **SYK** | **TCF3** | **TINF2** |
| **TRAF3** | **TSR2** | **UBE2T** | **USB1** | **WRAP53** | **XRCC1** | **XRCC2** |  |

33 leukemia fusion genes detected by RT-PCR in our study

| **AML1/ETO** | **PML/RARA** | **CBFβ/MYH11** | **MLL/AF4** |
| --- | --- | --- | --- |
| **SET/CAN** | **MLL/SEPT6** | **E2A/PBX1** | **TEL/AML1** |
| **SIL/TAL1** | **E2A/HIF** | **NPM/MLF1** | **PLZF/RARA** |
| **DEK/CAN** | **MLL/AF9** | **SET/NUP214** | **NPM/RARA** |
| **CALM/AF10** | **FIP1L1/PDGFRA** | **AML1/MTG16** | **TLS/ERG** |
| **NUP98/HOXA9** | **MLL/AF10** | **HOX11** | **HOX11L2** |
| **TEL/PDGFRB** | **MLL/AF1q** | **MLL/AF6** | **MLL/ENL** |
| **MLL/ELL** | **MLL/AF1p** | **MLL/AF17** | **BCR/ABL** |
| **MLL/AFX** |  |  |  |
